# A systematic review and meta-analysis on ketamine use and postoperative delirium in older patients undergoing spine or orthopedic surgery

**DOI:** 10.1007/s44254-025-00145-y

**Published:** 2026-01-05

**Authors:** Andrew Sretavan, Marc Buren, Peggy Tahir, Jacqueline M. Leung

**Affiliations:** University of California, Department of Anesthesia & Perioperative Care and UCSF Library, San Francisco, CA 94143-0648 USA

**Keywords:** Postoperative delirium, Ketamine, Spine, Orthopedic surgery

## Abstract

**Purpose:**

Postoperative delirium (POD) is common in older adults following major surgery. Ketamine has opioid-sparing properties and potential neuroprotective effects, yet whether intraoperative ketamine reduces the incidence of postoperative delirium remains uncertain. This systematic review aims to determine whether ketamine use is associated with a lower incidence of POD in older patients undergoing spine or orthopedic surgery.

**Methods:**

We conducted comprehensive searches in PubMed, Web of Science, EMBASE, Cochrane CENTRAL, and CINAHL Complete from inception to June 26, 2025. Both keywords and index terms (Mesh/EMTREE) were used to develop broad and sensitive searches tailored to each database to retrieve all relevant articles. EMBASE results were limited to articles and articles in press. Eligible studies included patients aged ≥ 60 years undergoing surgery with intraoperative ketamine administration and reporting POD as an outcome. Two reviewers independently screened studies, extracted data, assessed risk of bias, and performed quality appraisal using RoB 2 for randomized controlled trials (RCTs) and ROBINS-I for observational studies. Random-effects meta-analyses were performed to calculate odds ratios (ORs) with 95% confidence intervals (CIs).

**Results:**

We included 24 studies in the final analysis and five specifically studied patients undergoing spine or orthopedic surgery. The effect of ketamine on POD showed an OR of 1.50 (95% CI 0.38–5.91, *P* = 0.56 random effects model) for the two RCTs. Including the additional three cohort studies was marginally associated with an increase in the occurrence of postoperative delirium, OR 1.30 (95% CI 1.01–1.67), *P* = 0.0425. However, the imprecision of the studies was deemed to be serious due to small sample sizes, and only two were RCTs.

**Conclusions:**

This systematic review and meta-analysis did not find a statistically significant association between intraoperative ketamine use and POD in older patients undergoing spine or orthopedic surgery in the RCTs. However, because of the small number of available studies together with imprecision, this conclusion should only be considered as preliminary. Larger, high-quality randomized trials are needed to determine whether ketamine influences POD risk and to assess different dosing strategies for their effects on postoperative cognitive outcomes in older adults.

**Supplementary Information:**

The online version contains supplementary material available at 10.1007/s44254-025-00145-y.

## Introduction

Postoperative delirium (POD) appears especially prevalent after spine surgery and major hip or knee arthroplasty [1], driven in part by substantial postoperative pain—commonly managed with intravenous opioids, and the demographic profile of older patients who frequently undergo these surgeries. POD is a common and serious adverse event following major surgery in older patients, with reported incidence rates ranging from 10%–50% [1]. POD is characterized by an acute change in mental status, inattention, and fluctuating levels of consciousness. The occurrence of POD is associated with prolonged hospital stays, higher healthcare costs, and increased morbidity. Among non-cardiac procedures, opioids are known to cause mental status changes, even in healthy individuals [2], potentially exacerbating the risk of delirium. Moreover, inadequate pain control has been identified as an independent risk factor for the development of POD [3]. Studies have shown that patients experiencing higher levels of pain postoperatively are more likely to develop delirium [3], highlighting the importance of effective pain management strategies that minimize cognitive side effects.

Ketamine is a non-narcotic analgesic that exerts its effects primarily through antagonism of N-methyl-D-aspartate (NMDA) receptors, leading to inhibition of excitatory glutamate neurotransmission [4]. NMDA receptors play a critical role in synaptic plasticity, learning, and memory formation by regulating calcium influx and modulating neuronal communication. While ketamine-induced NMDA receptor blockade may disrupt normal cognitive processes, which could influence the development of POD, it may also confer neuroprotective benefits by attenuating excitotoxic neuronal damage from excessive glutamate release during surgical stress. Furthermore, ketamine’s rapid-acting antidepressant properties, likely mediated by NMDA receptor modulation and enhanced synaptic connectivity [5], could theoretically reduce delirium risk among patients with baseline mood disorders, as depression is an established risk factor for POD. While there is convincing evidence that ketamine is opioid-sparing, conflicting results have been reported about its direct effect on POD. Although a reduction in the use of postoperative opioids should be salutary given the adverse effects of opioids on cognition, whether ketamine use results in a lower incidence of POD has not been definitively demonstrated.

Spine surgery and orthopedic surgery such as arthroplasty of the hips and knees are among the most common surgical interventions performed in older adults; over 700,000 of these operations took place in the United States in 2018 alone [6]. As the population ages, demand for spine procedures has risen sharply [7], and the median age of patients undergoing spine surgery has steadily increased over the past two decades [7, 8]. Likewise, the average age of those undergoing hip or knee arthroplasty is 65–66 years [9] reflecting the prevalence of age-related spine and major joints disorders in this population. These surgeries are often undertaken to manage degenerative conditions such as osteoporosis and osteoarthritis that can cause chronic pain, loss of mobility, and diminished quality of life. Despite the potential benefits of surgical intervention, older adults undergoing these procedures face heightened risks of postoperative adverse events, including delirium. The multifactorial pathogenesis of POD, which encompasses surgical stress, pain management strategies, medication effects, and preexisting vulnerabilities, underscores the importance of identifying interventions that can reduce the incidence of this condition in a high-risk population.

Accordingly, we conducted a systematic review to assess whether the use of ketamine in older patients undergoing spine or orthopedic surgery is associated with a reduced incidence of POD. By summarizing existing literature, we aimed to evaluate if ketamine affected the risk of POD. We hypothesized that patients undergoing the above procedures who have received ketamine during surgery would experience a decreased incidence of POD.

## Methods

This review followed the guidelines outlined in the Preferred Reporting Items for Systematic Review and Meta-Analysis Protocol (PRISMA-P) [10], and was registered in the International Prospective Register of Systematic Reviews (PROSPERO; registration no. CRD420251024716). Our initial aim was to target spine and major arthroplasty surgery. However, since many studies did not report the specific type of orthopedic surgery, we modified our aim to be inclusive, to include the term orthopedic surgery rather than major arthroplasty surgery only.

### Eligibility criteria

Eligible studies included randomized controlled trials (RCTs), prospective or cohort studies, and case control studies for patients undergoing surgery. We included studies in which patients were aged ≥ 60 years and received ketamine or S-ketamine during the intraoperative period. Our pre-specified primary outcome was POD. No restrictions were placed on language, date of publication, or type of surgery. After the initial search, we amended the search in June 2025 to include S-ketamine and additional search terms.

### Search strategy

With the assistance of a research librarian (PT), we conducted comprehensive literature searches using the PubMed, Web of Science, Embase, Cochrane CENTRAL, and CINAHL Complete databases. Searches were developed using both index terms (Mesh/Emtree) and keywords, depending on the search parameters for the specific database. The searches were broad and sensitive, using multiple synonyms for each concept, to ensure we did not miss any important articles. The developed searches centered on the concepts of delirium, ketamine, surgery or the postoperative period, and the elder population. We did not impose any date restrictions, though we limited our Embase results to articles and articles in press, and we limited the CINAHL results to peer-reviewed articles. The full search strategies for each database can be found in the search appendix (Supplementary Material 1). We approached the gray literature by developing several Google searches to find reports, clinical guidelines or other authoritative and supporting information.

The specific search terms for each database are included in Supplementary Material 2.

### Selection process

Two reviewers independently screened titles and abstracts in duplicate (AS and JML). We retrieved the full text of all potentially relevant studies. The full text of these studies was retrieved and read in duplicate; those that fulfilled the selection criteria were included in the review. Disagreements were resolved by consultation with a third reviewer (MB), and agreement on discordant decisions was reached by consensus.

### Data extraction

References were downloaded with Endnote 21 and transferred to Covidence (Covidence systematic review software. Melbourne, Australia. Veritas Health Innovation) for screening and data extraction.

For data extraction, two reviewers extracted the data using the standardized data extraction form on Covidence which detailed the following:

- General characteristics: first author, year and journal of publication, language, countries, study design (RCTs, prospective or retrospective cohort studies).
- Patient characteristics: main eligibility criteria, age, sex, type of surgery.
- Intervention: whether ketamine was used, including the method and timing of administration, if available.
- Methods – how the primary outcome, POD was measured.
- Results: for each group, the number of events and number of patients analyzed for binary outcomes were recorded. Odds ratios (OR) and 95% confidence intervals (CIs) were extracted. If an OR was not recorded, we computed the OR using incidence of POD provided in the publication. We also measured if the estimates for cohort studies accounted for the presence of co-variates.

### Quality of study assessment

Two reviewers independently assessed study quality and evidence. We performed study quality assessments using RoB 2 (a revised tool for assessing risk of bias in randomised trials) for RCTs [11] and ROBINS-I (Risk of Bias in Non-randomized Studies of Interventions) for observational studies [12].

### Statistical analysis

Meta-analysis was conducted using R, version 4.5.1 (2025–06–13; R Foundation for Statistical Computing, Vienna, Austria) and the meta package version 8.2–1. Analyses used OR as the effect size measured with 95% CI, significance was set at *P* < 0.05, with the study as the unit of analysis. We built forest plots for the primary outcome using the random effect meta-analysis command. We performed subgroup analyses for POD according to the study design, methods to measure POD, prospective vs. retrospective nature of the study, and sample size. We used funnel plots to evaluate the presence of small study effects. We assessed heterogeneity using meta regression, and publication bias using the funnel plot and the Egger’s test. We performed sensitivity analysis using the leave-one-method. To quantify unmeasured confounding factors (data gap), we performed sensitivity analysis using the E-value method.

## Results

### Systematic review results

**Fig. 1:**
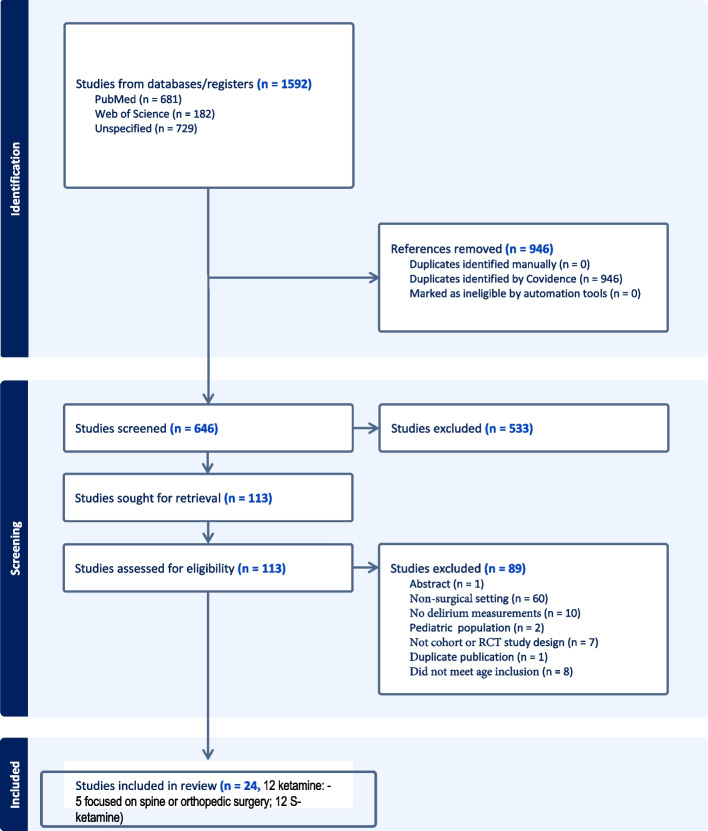
Flow diagram of the study selection process

We identified a total of 1,592 studies which were imported to Covidence for screening. After removing duplicates (946), and those not meeting inclusion criteria (533), 113 studies remained to be screened for final eligibility (Fig. 1). After full text review, a total of 24 studies remained in the final analysis. Common reasons for exclusion included patient age < 60 years, absence of POD as an outcome, lack of intraoperative ketamine use, or non-surgical populations. Among the remaining 24 studies, we performed additional full text review to ensure that the primary outcome, age criterion (60 years or older) and study setting met the review inclusion criteria. Of the 24 studies, 12 involved ketamine and 12 involved S-ketamine. Of the 12 studies involving racemic ketamine, only five focused on either spine or orthopedic surgery [13–17].

### Primary outcome on studies involving the use of ketamine

Of the five studies that focused on patients undergoing spine or arthroplasty surgery, two were randomized, placebo-controlled trials [13, 14] and two were prospective cohort studies [15, 16], with the remaining one being a retrospective cohort study [17]. Only two of the three cohort studies accounted for co-variates in determining the estimates [16, 17]. A summary of the included studies appears in Table 1.

**Table 1:** Summary of the systematic review of the patients undergoing orthopedic or spine surgery

Author	Year	Study Design	Sample size	Patient types	Intervention	Timing of ketamine administration	Methods of ketamine administration	Delirium measurements
Ma et al. [13]	2013	RCT	120	Elective orthopedic surgery	Randomly divided into 4 groups of 30 patients each: normal saline (control group), ketamine group, dexmedetomidine group, ketamine + dexmedetomidine group	Before anesthesia induction	Ketamine 0.5 mg/kg bolus	CAM 1 h after surgery, postoperative days 1 and 3
Weinstein et al. [17]	2018	Retrospective cohort Estimates adjusted for co-variates	41,766	Total knee and hip arthroplasty	None	NA	NA	ICD-9 code from chart review
Barreto Chang et al. [15]	2022	Prospective cohort Estimates not adjusted for co-variates	155	Elective spine surgery	None	NA	NA	Nu-DESC and CAM-ICU, timing not specified
Tekletsadik et al. [16]	2024	Prospective cohort Estimates adjusted for co-variates	220	Elective orthopedic surgery	None	Anesthesia induction	NA	CAM daily for first three postoperative days
Verdonk et al. [14]	2024	RCT	292	Major orthopedic surgery	Randomly assigned in a 1:1 ratio to receive preoperative ketamine 0.5 mg/kg as an intravenous bolus (*n* = 152) or placebo (*n* = 149) in random blocks stratified according to the study site	Preoperative	Ketamine 0.5 mg/kg bolus	CAM twice daily from 2 h after surgery to postoperative day 7

The detailed reference of each study can be found in the manuscript text

*CAM-ICU* confusion assessment method for the intensive care unit, *ICD-9* international classification of diseases, ninth revision, *Nu-DESC* nursing delirium screening scale, RCT randomized controlled trial

**Fig. 2:**
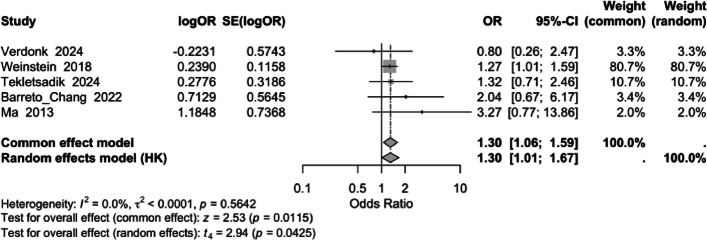
Forest plot summarizing the meta-analysis of five studies involving patients undergoing spine or orthopedic surgery. Each study’s estimate is represented by a square, with the size proportional to the study’s weight, which is based on the precision of its estimate. The vertical line indicates no effect. Individual estimates are pooled to produce an overall estimate, shown as a black diamond, where the center represents the pooled effect and the width represents the 95% confidence interval. The pooled estimate is also presented numerically

Our meta-analysis demonstrated a consistent effect across studies, with no evidence of heterogeneity (${I}^{2}=0\text{\%}$). Excluding the cohort studies, the effect of ketamine on POD showed an OR of 1.50 (95% CI 0.38–5.91, *P* = 0.56) using a random-effects model; and an OR of 1.36 (95% CI 0.56–3.31, *P* = 0.495) using a fixed-effects model, for the two RCTs. The pooled estimate under a fixed-effect model for all five studies showed that the intervention was associated with a 30% increase in the odds of the outcome (OR = 1.30, 95% CI: 1.06–1.59, *P* = 0.0115). This effect remained significant, though less robust, under a random-effects model (OR = 1.30, 95% CI: 1.01–1.67, *P* = 0.0425). These findings suggest a modest but consistent association. However, the conclusions are tempered by the relatively small number of included studies and the possibility of publication bias, which could influence the observed effect size. A forest plot of the results is shown in Fig. 2. The two RCTs utilized a single bolus of ketamine at 0.5 mg/kg before anesthetic induction [13, 14], while the other non-randomized studies did not measure the dose or timing of ketamine administration [15–17].

### Heterogeneity and publication bias analysis

The funnel plot and the Egger test did not show any evidence of publication bias or small studies effect. We conducted a meta-regression to analyze if study heterogeneity was because of the study population size, methods of delirium analysis, study design (randomized *vs*. cohort), and whether the study was prospective *vs*. retrospective. Our analyses did not show significant evidence for heterogeneity owing to any of these variables.

### Impact of surgery type and study design on reported results

**Fig. 3:**
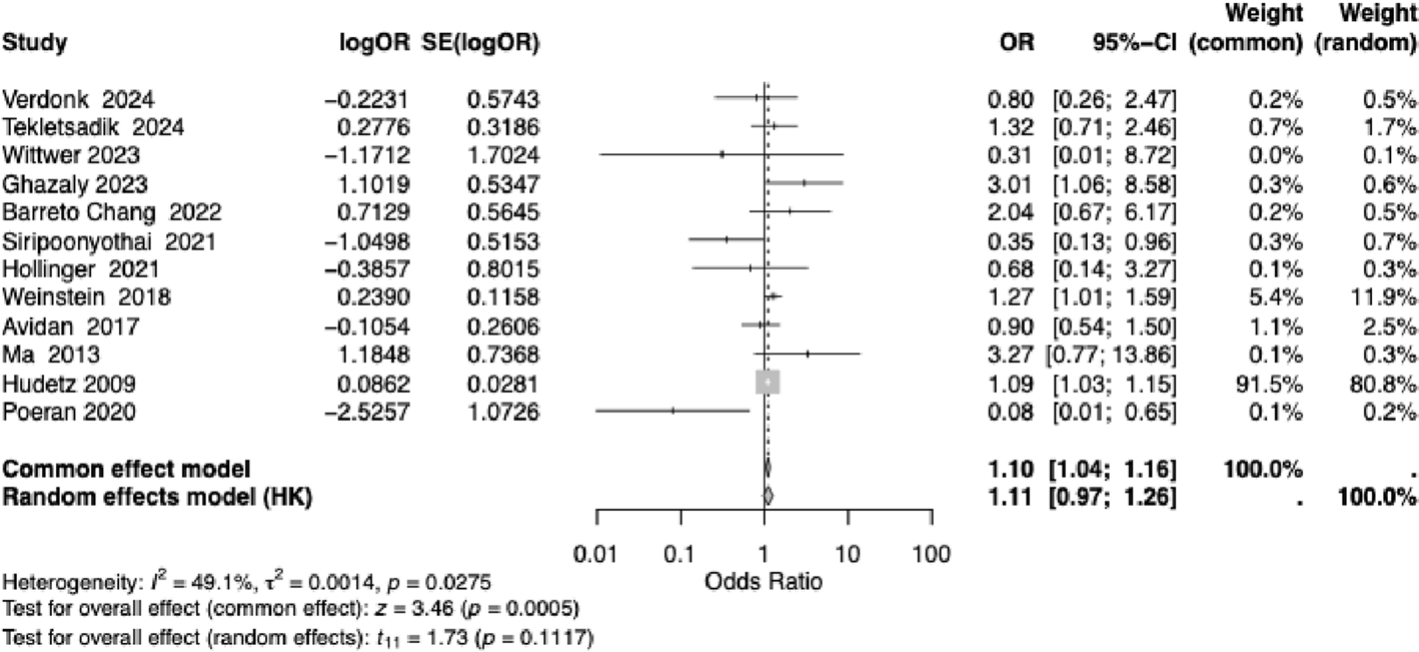
Forest plot summarizing the meta-analysis of 12 studies, including 7 studies that did not involve spine or orthopedic surgery

To assess whether the effect of ketamine on POD differed by surgical type, we explored whether including other types of surgery (cardiac, non-spine and non-orthopedic) influenced the results. We pooled the additional seven studies and performed additional meta-analysis. Across the 12 included studies, there was moderate heterogeneity (*I*^2^ = 49.1%, *P* = 0.0275). The pooled OR under a fixed-effect model indicated a small but statistically significant increase in odds (OR = 1.10, 95% CI: 1.04–1.16, *P* = 0.0005). However, when accounting for between-study variability using a random-effects model, the effect was attenuated and no longer statistically significant (OR = 1.11, 95% CI: 0.97–1.26, *P* = 0.1117, Fig. 3). These findings suggest a possible modest association, but the presence of heterogeneity indicates that the studies differ substantially in their results which calls for caution in interpretation.

Using meta-regression, we further determined that non-randomized studies had high between study variance (*r*^*2*^ = 0.87), and very high heterogeneity *I*^*2*^ = 90.84%). Heterogeneity within the group is statistically significant (*P* < 0.001). In contrast, for the RCTs, the between study variance was low (*r*^*2*^ = 0.05), but showed moderate heterogeneity across all studies (*I*^*2*^ = 68.25%). The overall meta-analysis for between-group differences showed no significant difference between the RCTs and the cohort studies (Qb(1) = 0.73, *P* = 0.39).

### Postoperative delirium measurements

Assessment tools for POD varied across studies. Most used the Confusion Assessment Method (CAM), although one employed both Nu-DESC (Nursing Delirium Screening Scale) and CAM-ICU (Confusion Assessment Method for the Intensive Care Unit) [15], and another diagnosed delirium based on ICD-9 codes from chart review [17]. The measurements of delirium were dichotomous. One study performed sub-group analysis [15] and found that patients with preoperative cognitive impairment who had received intraoperative ketamine had higher rate of POD, but the confidence interval of the estimate was very wide suggesting the uncertainty of the estimate.

We conducted a meta-regression analysis stratifying the five studies on spine or arthroplasty surgery into measurement of delirium by CAM *vs*. other methods. The results showed that there was no statistically significant difference between measurements of delirium by CAM *vs*. other methods. All *τ*^*2*^ and *I*^*2*^ = 0 suggesting that studies were very consistent within and across groups. The grouping by methods of delirium analysis did not significantly impact effect size (*P* = 0.65). However, the study by Weinstein et al*.* [17]*,* dominated the analysis because of its sample size which may have influenced the pooled effect size. However, the leaveoneout analysis shows that the meta-analysis result was robust, and no single study drastically changes the overall pool estimate (*I*^*2*^ = 0, *P* = 0.47).

### Quality and evidence assessment of the selected studies

The assessments using RoB 2 for RCTs and ROBINS-I for observational studies showed low to moderate bias (Tables 2 and 3).

**Table 2:** Risk of bias in randomized studies assessed using the RoB 2 tool

**Bias Domain**	**Ma et al**	**Verdonk et al**
Randomization process	Low	Low
Deviation from intended interventions	NI	Low
Missing outcome data	NI	NI
Measurement of the outcome	Mod	Low
Selection of the reported Results	Mod	Low
Overall bias: low/moderate/serious/critical/NI	Mod	Low

*NI* no information

**Table 3:** Risk of bias in nonrandomized studies assessed using the ROBINS-I tool

Bias Domain	Tekletsadik	Barreto Chang	Weinstein
Bias because of confounding	Low	Moderate	Low
Bias in selection of participants into the study	Low	Moderate	Low
Bias in classification of interventions	Low	Low	Low
Bias because of deviations from intended interventions	NI	NI	NI
Bias because of missing data	NI	NI	Moderate
Bias in measurement of outcomes	Low	Moderate	Moderate
Bias in selection of reported results	Low	Moderate	Low to moderate
Overall bias: low/moderate/serious/critical/NI	Low	Moderate	Low to moderate

*NI* no information

### Role of unmeasured confounding variables

To quantify unmeasured confounding factors (data gap), we performed sensitivity analysis using the E-value method [18]. For the five studies on spine or orthopedic surgery, the E- value was calculated to be 1.76. The relatively small E-value suggests that even a modest confounder could potentially explain away the association. Therefore, the observed association is not particularly robust to unmeasured confounding.

### S-ketamine summary

A comprehensive review of S-ketamine is beyond this meta-analysis which focused on the use of ketamine. However, we did included the keyword S-ketamine in the search and identified 12 studies that met the age inclusion criterion of 60 years or older undergoing surgery [19–30] (Table 4). Only three of the 12 studies focused on patients undergoing orthopedic surgery [21, 25, 29], and only one reported that esketamine anesthesia reduced incidence of POD [21].

**Table 4:** Summary of S-ketamine studies

Author	Year	Setting / Surgery Type	Design	Esketamine Dose	Main Outcomes	Key Findings / Conclusion
Bornemann-Cimenti et al. [19]	2016	Major abdominal surgery	Triple-blind RCT, *N* = 90	Placebo vs. minimal dose (0.015 mg/kg/h) vs. low-dose (0.25 mg/kg bolus, 0.125 mg/kg/h)	Opioid use, delirium, hyperalgesia	Minimal-dose reduced opioids & hyperalgesia with fewer delirium events
Ding et al. [20]	2024	Breast cancer surgery	RCT, *N* = 100	0.25 mg/kg bolus + 0.125 mg/kg/h	Opioid use, cognition, satisfaction	No difference in delirium. Ketamine group had better cognition, used less opioids, and had less hypotension/tachycardia, and better satisfaction
Hua et al. [21]	2025	Hip replacement	RCT, *N* = 114	Opioid free Anesthesia with Esketamine- vs. Fentanyl/remifentanil	Delirium (CAM-CR)	Esketamine-only anesthesia reduced POD significantly
Liu et al. [22]	2024	GI surgery	RCT, *N* = 60	Esketamine- (1 mg/kg) + low-dose sufentanil vs. full-dose sufentanil	NRS pain, IL-6, POD	Esketamine- group had less pain, lower IL-6, and fewer delirium cases
Lu et al. [23]	2024	Thoracic anesthesia	RCT, *N* = 94	0.5 mg/kg IV Esketamine- vs. dexmedetomidine	Pain (VAS), sleep, cognition	Esketamine superior to Dex for pain, mood, hemodynamics, and delirium
Luo et al. [24]	2024	Non-cardiac thoracic surgery	RCT, *N* = 129	0.2 or 0.5 mg/kg (single dose)	HADS, VAS, biomarkers	No difference in POD between any group. 0.5 mg/kg reduced anxiety, depression, pain and inflammatory markers vs. other groups
Ma CB. et al. [25]	2024	Total hip/knee arthroplasty	RCT, *N* = 260	0.2 mg/kg + infusion (0.125 mg/kg/h)	POD, pain, sleep, hemodynamics	No POD difference, but better pain & hemodynamics with Esketamine superior to group
Ma JM. et al. [26]	2023	GI tumor surgery	RCT, *N* = 68	0.25 mg/kg + 0.125 mg/kg/h infusion	DNR, POD, opioids	No difference in POD. Lower DNR, better hemodynamics, less opioid use, lower pain in the esketamine group
Wang M. et al. [27]	2024	Non-cardiac elderly surgery	RCT, *N* = 124	0.5 mg/kg IV	Cognitive tests, POD, vitals	No difference in delirium. Less cognitive dysfunction, better BP stability, no increase in side effects
Wang Y. et al. [28]	2024	Thoracic surgery (elderly)	Retrospective cohort, *N* = 140	Low-dose S-ketamine vs. none	POD, POCD, cognition, side effects	Lower POD and POCD; improved cognition; no increased side effects
Ye et al. [29]	2024	surgery (Elective hip geriatric)	RCT, *N* = 121	Esketamine + dexmedetomidine (opioid-free anesthesia) vs. opioid anesthesia	Composite complications, pain, hemodynamics, delirium	Reduced complications (nausea, vomiting, hypoxemia); better hemodynamics; less motion pain; no difference in POD
Zhang et al. [30]	2024 2024	Lung cancer surgery	RCT, *N* = 172	Esketamine + Dex vs. Dex only	POD, QoR-15, immunity markers	POD halved, better recovery, immune & anti-inflammatory benefits

*CAM* confusion assessment method, *CAM-CR* confusion assessment method–chinese revision, *Dex* Dexmedetomidine, *DNR* delayed neurocognitive recovery, *HADS* hospital anxiety and depression scale, *IL-6* Interleukin-6, *NRS* numeric rating scale, *POD* postoperative delirium, *POCD* postoperative cognitive dysfunction, *QoR* quality of recovery, *RCT* randomized controlled trial, *VAS* visual analog scale

## Discussion

Our systematic review and meta-analysis found that based on the two RCTs in patients who have undergone spine or major orthopedic surgery, the effect of ketamine on POD was not significant. However, this conclusion was limited by only two studies of randomized controlled study design. Including the three cohort studies shows a small increase in the odds of POD. When the analysis was broadened to include studies of other surgical types, ketamine was found to be not associated with an increased incidence of POD. However, this secondary finding is limited by substantial heterogeneity, which constrains its interpretability and clinical applicability. Most importantly, our review identified that there was insufficient high quality, randomized clinical trials targeting the highest risk patients, especially those with preoperative cognitive impairment. In addition, most studies focused on single bolus of ketamine administration, and none had investigated the continuous infusion during and after surgery.

### Comparison with previous studies

Our review differs from prior meta-analyses in three important ways. First, we focused on the older patients who have a higher baseline risk to develop POD. Second, we focused on spine and major orthopedic surgery including joint replacement surgeries, because patients undergoing these procedures may have pre-existing pain and the procedures are particularly painful, frequently necessitating opioids. Third, we separately evaluated racemic ketamine and S-ketamine.

Ketamine is known to be opioid sparing, and theoretically, decreasing opioid use might help reduce adverse cognitive outcomes, including delirium. However, our results with the limited studies available do not support this hypothesis: across the limited studies available, ketamine did not demonstrate a clear benefit in lowering delirium incidence, and some studies even hinted at a potential increase in delirium risk, albeit with methodologic limitations [15, 16]. Notably, these two studies were non-randomized, and in one of these two, only patients with preoperative cognitive impairment showed a higher incidence of POD, and the confidence interval was large, suggesting the imprecise nature of the estimate and complicating any definitive conclusions [15].

Even when limiting the analysis to randomized trials, no protective effect of ketamine was observed [13, 14]. Moreover, the mode and timing of ketamine administration was typically limited to only a single bolus before anesthetic induction [14, 31]. Given the pharmacokinetics of ketamine where a rapid onset of action is achieved with intravenous administration, achieving peak plasma concentrations and redistribution from the central nervous system to peripheral tissues occur quickly, it is not surprising that a single bolus of ketamine without continuous infusion would not have substantial impact on POD. Postoperative ketamine infusions, which might prolong analgesic or neuroprotective effects, have not been systematically studied. Finally, none of the reviewed studies focused on the highest risk patients, especially those with prior cognitive impairment. Therefore, the lack of association with POD from the current meta-analysis reflects the serious limitation of indirectness of the available studies.

Prior meta-analyses investigating the association of ketamine and POD showed conflicting results (Table 5) [32–41]. Most importantly, there is substantial heterogeneity of the studies being reviewed. Only a few prior meta-analysis reviews focused on the older patients [33, 34, 37, 39, 41]. Most meta-analyses concluded that there was no difference in the incidence of POD in patients who received ketamine *vs*. those who did not [33, 35, 37, 39, 41]. Of the ones that showed a difference [32, 34, 36, 40, 42], there appears to be a beneficial effect of ketamine although the studies were heterogeneous in terms of the types of surgery included, comparison group not being placebo [32], use of ketamine combined with another agent [34], or the primary outcome being a composite including not only delirium [42]. None of these meta-analysis reports focused on patients undergoing only spine or orthopedic surgery, thus a direct comparison with our present findings is not possible. Finally, none of the reviews distinguished between single bolus *vs*. continuous infusion of ketamine. Therefore, previous meta-analyses show mixed results in the effect of ketamine on POD.

**Table 5:** Previous meta-analysis summary

Author	Year	Setting	No of studies	Findings
Fellous et al. [39]	2023	Perioperative ketamine administration in adult patients compared to placebo or no intervention	14	OR 0.93, 95% CI 0.51–1.70 Perioperative ketamine does not prevent postoperative delirium
Viderman et al. [41]	2023	Meta-analysis of RCTs comparing ketamine use (experimental group) with placebo (controls)	8	OR 1.03, 95% CI 0.61–1.73, no statistically significant difference in incidences of POD between the ketamine and control groups
Meco* et al. [40]	2023	RCTs on pharmacological prevention of POD in patients undergoing cardiac surgery	22	OR 0.05, 95% CI 0.00–0.86, ketamine was effective in preventing POD in cardiac surgery patients
Reisinger et al. [37]	2023	RCTs and prospective observational studies, and nested case–control studies in patients aged > 65 years in medical, surgical or ICU patients	31	OR 0.72, 95% CI 0.35–1.46, no increased delirium risk for ketamine
Lee* et al. [34]	2021	RCTs comparing two or more pharmacological interventions, including placebo, to prevent or reduce POD in patients receiving surgery under general or regional anesthesia	51	OR -2.72, 95% CI −5.80–0.36, the incidence of POD decreased by 86% in combination of ketamine and dexmedetomidine
Wang* et al. [38]	2020	RCTs of analgesic effect of pre op ketamine versus a control for patients undergoing THA or TKAs	21	Statistically lower OR was observed in the ketamine group than that in the control group after the pooled analysis (OR = 0.54, 95% CI 0.37–0.77, *P* = .0008). But this study combined delirium with other “side effects”
Cui* et al. [32]	2020	Double blinded RCTs comparing sedative/anesthetic agents	39	OR 0.3, 95 CI 0.09–0.99, compared to midazolam in association with POD
Liu et al. [35]	2019	Systematic Review of RCTs investigating pharmacological prevention of POD in adults	38	Risk Ratio 0.48, 95% CI 0.07–3.13
Hovaguimian et al. [33]	2018	Metaanalysis effects of intraoperative ketamine administration RCT	6	OR 0.83, 95 CI 0.25–2.80 Incidence of post op delirium did not differ significantly between groups
Mu* [36]	2015	RCTs of various pharmacologic agents on prevention of POD	7	OR 0.11, 95 CI 0.02–0.82, suggesting potential benefit of ketamine in preventing POD

*CI* confidence interval, *OR* = odds ratio, *POD* postoperative delirium, *RCT* randomized controlled trial, *THA* total hip arthroplasty, *TKA* total knee arthroplasty

^*^ Denotes significant difference between ketamine and control groups

Esketamine has recently emerged as a favored treatment option, particularly for its rapid-acting antidepressant effects in treatment-resistant depression, and is commonly administered via an intranasal route [43]. S-ketamine represents the S-enantiomer of ketamine with a markedly higher affinity for the NMDA receptor and distinct pharmacokinetic and pharmacodynamic properties compared to the racemic mixture. These differences may result in variable effects on neuronal activity and postoperative neurocognitive outcomes, including delirium. Although S-ketamine offers non-invasive administration [44], its availability varies significantly by region: In the United States, only racemic ketamine is approved for intravenous surgical use, while esketamine is restricted to intranasal depression treatment. Conversely, European and Asian countries permit intravenous esketamine for anesthesia, where it demonstrates comparable intraoperative hemodynamic stability to racemic ketamine but with potentially reduced emergence phenomena.

Our review showed few studies focused on patients undergoing spine or orthopedic surgery. Future research should directly compare the effects of esketamine and racemic ketamine to better understand their respective efficacy and safety profiles in the context of POD.

### Potential limitations

This meta-analysis should be interpreted considering several limitations. First, the number of available studies was relatively small, which limits statistical power and the ability to explore potential sources of heterogeneity. Second, although statistical tests did not indicate heterogeneity, subtle differences in study populations, interventions, and outcome definitions may still influence the pooled effect estimate. Third, we could not formally exclude the presence of publication bias, as the small sample size limited the reliability of funnel plot–based assessments. Finally, variations in study quality and reporting standards may have introduced residual confounding.

When we introduced the additional seven studies into the overall model, there was moderate heterogeneity and limited precision of several included studies, therefore the overall evidence remains inconclusive. Larger, well-designed studies will be essential to determine whether the observed trend reflects a true effect or study-level variability.

In addition, although we performed a comprehensive literature search in all relevant data bases, our approach is limited to available published studies and the reported results may be skewed by publication bias, since studies with positive results are more likely to be published, leading to an overestimation of treatment effects. The validity of the included studies such as lack of randomization can affect the reliability of the results. We addressed this limitation using sub-group analysis by excluding non-randomized controlled studies. However, only two studies were RCTs and risk of bias was deemed to be low to moderate. Therefore, the results should be interpreted with caution. In addition, given that there were only five studies which focused on orthopedic or spine surgery, Egger’s test is underpowered. Publication bias cannot be reliably assessed.

### Clinical implications

Our review of prior studies cumulatively suggests that a single bolus of ketamine appears not to increase the occurrence of POD in patients undergoing spine or orthopedic surgery in the two RCTs. However, we cannot extrapolate this finding to patients with preoperative cognitive impairment undergoing spine or major orthopedic surgery as there was insufficient information to address this vulnerable population. Our review is consistent with the fact that there is currently no existing guidelines or definitive recommendation to suggest the routine use or non-use of prophylactic ketamine in decreasing POD [45, 46]. Further evaluation of use of intra- and post-operative ketamine infusion in limiting the use of intra- or post-operative opioids to limit POD based on its opioid sparing effect has not been investigated.

An important observation resulting from our comprehensive review shows that there is still a significant gap in understanding the role of perioperative use of ketamine in the older subjects with respect to postoperative pain control and cognition because of the lack of high quality, and large RCTs in the at-risk older subjects. Therefore, concluding whether ketamine should be avoided or used in these patients is premature.

### Conclusions

This systematic review and meta-analysis found that a single dose of intraoperative ketamine appears not to increase the incidence of POD in older adults undergoing spine or orthopedic surgery in RCTs. Including the three cohort studies showed a slight increased odds of POD. While broader analyses across all surgical types did not show protective or deleterious effects, these results are confounded by heterogeneity and study design limitations. The major limitation from prior studies is that they did not focus on the highest risk patients such as those with prior cognitive impairment or those with substantial perioperative pain, and ketamine use was only limited typically to a single bolus prior to anesthetic induction. Therefore, future trials should prioritize these high-risk groups and evaluate both bolus and infusion strategies in rigorously designed, placebo-controlled studies.

This meta-analysis should be considered as exploratory, and the results should be validated when more prospective RCTs become available.

## Supplementary Information

Supplementary Material 1.

Supplementary Material 2.

## Data Availability

Not applicable.

## Abbreviations

CAM: Confusion assessment method
CI: Confidence interval
NMDA: N-methyl-D-aspartate
OR: Odds ratio
POD: Postoperative delirium
RCT: Randomized controlled trial
ROB: Risk of bias
