# Supplementary material for: A systematic review and meta-analysis on ketamine use and postoperative delirium in older patients undergoing spine or orthopedic surgery
Source: Anesthesiol Perioper Sci. 2026 Jan 5;4(1):2. doi: 10.1007/s44254-025-00145-y (PMC12765724; doi:10.1007/s44254-025-00145-y)
Supplement: Supplementary file 1 — Supplementary Material 1. [file 44254_2025_145_MOESM1_ESM.docx]

**Supplementary table 1**

Search appendix – delirium and ketamine

| DATABASE | SEARCH STRATEGY |
| --- | --- |
|  |  |
| PubMed | (delirium OR "Delirium"[Mesh]) AND (ketamine OR "Ketamine"[Mesh] OR ketanest OR ketalar OR  esketamine OR "Esketamine" [Supplementary Concept] OR calipsol OR calypsol OR kalipsol OR ketaset) AND (surgery OR "surgery" [Subheading] OR “surgical procedure” OR “surgical procedures” OR "Surgical Procedures, Operative"[Mesh] OR "General Surgery"[Mesh] OR postoperative OR post-operative OR "Postoperative Period"[Mesh]) AND (elder OR elders OR elderly OR aged OR “Aged”[Mesh] OR “senior citizens” OR geriatric) |
| Web of Science | delirium AND (ketamine OR ketanest OR ketalar OR esketamine OR calipsol OR calypsol OR kalipsol OR ketaset) AND (surgery OR “surgical procedure” OR “surgical procedures” OR postoperative OR post-operative) AND (elder OR elders OR elderly OR aged OR “senior citizens” OR geriatric) |
| Embase | ('delirium' OR 'delirium'/exp) AND ('ketamine'/exp OR ketamine OR 'ketanest'/exp OR ketanest OR 'ketalar'/exp OR ketalar OR 'esketamine'/exp OR esketamine OR 'calipsol'/exp OR calipsol OR 'calypsol'/exp OR calypsol OR 'kalipsol'/exp OR kalipsol OR 'ketaset'/exp OR ketaset) AND ('surgery' OR 'surgical procedure' OR 'surgical procedures' OR 'surgery'/exp OR 'general surgery'/exp OR 'postoperative' OR 'post-operative' OR 'postoperative period'/exp) AND ('elder' OR 'elders' OR 'elderly' OR 'aged' OR 'aged'/exp OR 'senior citizens' OR 'geriatric') AND ('article'/it OR 'article in press'/it) |
| Cochrane CENRAL | delirium AND (ketamine OR ketanest OR ketalar OR esketamine OR calipsol OR calypsol OR kalipsol OR ketaset) AND (surgery OR “surgical procedure” OR “surgical procedures” OR postoperative OR post-operative) AND (elder OR elders OR elderly OR aged OR “senior citizens” OR geriatric) |
| CINAHL Complete | delirium AND (ketamine OR ketanest OR ketalar OR esketamine OR calipsol OR calypsol OR kalipsol OR ketaset) AND (surgery OR “surgical procedure” OR “surgical procedures” OR postoperative OR post-operative) AND (elder OR elders OR elderly OR aged OR “senior citizens” OR geriatric) |
